# Centering Care as Normal and Valuable

**DOI:** 10.1002/hast.70036

**Published:** 2025-12-11

**Authors:** Devan Stahl

**Keywords:** disability bioethics, empirical bioethics, clinical ethics, justice, medical decision‐making

## Abstract

*This commentary, which responds to the article “Models of Relational Medical Decision‐Making: Caregivers and Advanced Life‐Sustaining Treatment,” by Aaron Wightman and Georgina Campelia, in the same issue of this journal, argues for reorienting medical decision‐making around care rather than patient autonomy alone. Drawing on empirical studies and disability bioethics, it asserts that most patients prefer involving physicians and caregivers in decision‐making. Empirical studies show that many patients prefer physician‐directed or collaborative decisions and commonly weigh the input of caregivers. Additionally, patients frequently factor the potential burden on family into difficult treatment choices, undermining strict substituted decision‐making. Normatively, focusing on care acknowledges human dependency and the moral significance of caregiving, which resists ableist tendencies that render carers invisible. The author endorses Wightman and Campelia's care‐centered model, showing that it better reflects actual decision‐making practices and validates dependence and caregivers’ labor*.

## Other Voices

Many, if not most, bioethical accounts of medical decision‐making rest upon a dangerous fiction. Unless proven otherwise, bioethicists tend to assume that patients are rational, autonomous individuals who make decisions that promote their personal interests, goals, and values. This fiction accords with prominent Western philosophical values such as individualism, self‐determination, and independence, and it is clinically useful, as it helps clinical ethicists and clinicians focus their energies on shared decision‐making between the patient and physician. Yet this fiction of the autonomous individual fails to appreciate people's relational entanglements and moral obligations. In reality, few of us make important decisions without consulting others, particularly those who will be affected by our decisions. As Aaron Wightman and Georgina Campelia have convincingly argued in an article in this issue of the *Hastings Center Report*, caregivers are often treated unjustly when they are mere afterthoughts in the medical decision‐making process.^1^ The labors of caregiving can be joyful, burdensome, enriching, or draining (and are most often a combination of all these things), but they are not insignificant, nor are they perceived as insignificant to patients.

The common framing of medical decision‐making as an autonomous, individual endeavor is both empirically and normatively problematic. Wightman and Campelia have provided another model, the care‐centered model, that likely better represents how people make important decisions and how they *should* make those decisions. Rather than an achievement, it might be the sign of a life not well lived if a person made all their important medical decisions without input from or regard for people close to them. There remains room for the patient‐centered model that many of us have championed in bioethics, but when it comes to medical decisions of any consequence, Wightman and Campelia's model may be superior for a broader range of scenarios than they acknowledge. Rather than challenge their model, I will bolster it with empirical data and insights from my work in disability bioethics.

First, an empirical claim. Studies show that patients rarely want to make medical decisions alone. Population surveys reveal that most people want their physicians to offer them medical choices, but more than half prefer to leave all decisions about their medical care to their doctor.^2^ While such results may be surprising given how bioethicists often talk about self‐determination, the choice to let one's physician decide among treatment options still fits within the standard patient‐centered model of medical decision‐making described by Wightman and Campelia. The physician provides the patient with relevant medical information, the patient explores their own values and preferences, and the patient decides to accept the physician's recommended plan of care because they value their physician's knowledge and prefer to off‐load decision‐making to their physician.

A fault of some of these population studies is that they fail to account for other stakeholders in the medical decision‐making. When asked to consider the input of family members or loved ones, study respondents report that they also want to include these people in the decision‐making process. In a study of terminally ill patients, most (54 percent) said they want to make medical decisions in collaboration with their physicians, and 15 percent said they would delegate decision‐making responsibility to their physicians. At the same time, 44 percent of respondents said they would want to share medical decisions with their loved ones, and 6 percent indicated that they would rely completely on their loved ones for medical decision‐making. When asked whose opinion they would weigh the most, 51 percent of respondents said they would weigh both physicians’ and loved ones’ equally. Imagining a scenario in which they were unconscious, these same participants were more likely to weigh the input of their loved ones over that of their physicians.^3^ Even apart from the issue of caregiving by family members or friends, it is clear that few people desire to make important medical decisions alone.

The instinct to rely on others is bolstered when people imagine losing the capacity to make their own decisions. Bioethicists have stressed the importance of substituted decision‐making by surrogates, wherein the surrogate is asked to make the decisions the patient would have made. This becomes increasingly complicated, however, when we acknowledge that most patients do not want their surrogates to adhere strictly to their precedent decision‐making. In fact, few say they want their surrogates to practice pure substituted decision‐making.^4^ What most people say they want is for their treatment preferences to be weighed alongside input from their physicians and surrogates.^5^ Terminally ill patients consistently report that their care being “a burden on the family” is one of the most important factors in choosing among difficult treatment options.^6^ This last is very important for Campelia and Wightman's analysis; patients not only desire input from their surrogates when making difficult decisions, but they also make medical decisions with the burdens of their care in mind.

It is also possible to frame these responses within the patient‐centered model for medical decision‐making, insofar as a patient may request input from and consider the repercussions of their decisions on their loved ones, caretakers, or surrogates. Yet the patient‐centered model does not assume that the opinion of anyone except the patient should be given any weight beyond what the patient authorizes. Of course, it would be wrong to assume that all patients would want others’ input on their medical decisions, but those who do not may be the exception rather than the rule. Insofar as most patients do not want to become a burden on others, it seems only fitting that Wightman and Campelia's care‐centered model becomes the standard model when patients are faced with medical decisions that require substantial caretaking obligations from others. (Whether such caretaking would be perceived as a burden to the caretaker can be ascertained only in conversation with the caretaker.)

Second, a normative claim. Centering care in the decision‐making process not only validates many patients’ desire to involve others in the decision‐making process, but it also empowers all parties to acknowledge the truth about our human interdependence. This is a good thing. Bioethicists tend to center the rational, individual actor when discussing medical decision‐making, but by centering care, we acknowledge the dependence and caretaking that shape much of our lives. Our dependence on others, while present throughout the lifespan, is more salient when we are very young or old and when we become seriously ill. The introduction of advanced life‐saving technologies such as those described by Wightman and Campelia introduces yet another pivot point in which we may become especially reliant on others for our care or responsible for the intense care of another. One of the key lessons I have taken from disability studies is that dependence is neither shameful nor degrading. We all flourish when we learn to rely on one another and care for one another. Try as we might to avoid becoming dependent upon others or having others become dependent upon us, none of us can escape the obligations that come from being vulnerable, social beings.

Individual medical decisions, particularly significant ones, rarely affect only the individual. It would indeed be very odd, perhaps even morally wrong, for a patient to eschew or neglect every important person or obligation in their life when making a critical medical decision, particularly if their decision imposed significant caretaking obligations on another person. Oftentimes, the patient‐centered model and its companion, substituted decision‐making, perpetuate the false belief that most of us do, or even should, make decisions as isolated individuals, unconcerned with the views or effects the decision would have on others. This dangerous fiction not only perpetuates ableism; it also devalues the work of caretakers. As the disability philosopher Eva Feder Kittay argues, “[T]he denigration of care and dependency tends toward an attitude that makes the work and the value of the carers invisible, thus creating one oppression in the effort to alleviate another.”^7^ The care model articulated by Wightman and Campelia tends toward justice instead.
